# The taxonomic status and range distribution of *Boulenophrys shuichengensis* (Anura, Megophryidae)

**DOI:** 10.3897/BDJ.14.e188165

**Published:** 2026-04-07

**Authors:** Dongru Zhang, Lu Chen, Rongchuan Xiong, Wenjing Shen, Felista Kasyoka Kilunda, Weiwei Pan, Miao Zhong, Jianhong Li, Yunhe Wu

**Affiliations:** 1 School of Biological Science and Technology, Liupanshui Normal University, Liupanshui, China School of Biological Science and Technology, Liupanshui Normal University Liupanshui China; 2 Guizhou Academy of Forestry, Guiyang, China Guizhou Academy of Forestry Guiyang China; 3 Hubei Broad Nature Technology Service Co., Ltd., Wuhan, China Hubei Broad Nature Technology Service Co., Ltd. Wuhan China; 4 Yunnan Open University, Kunming, China Yunnan Open University Kunming China; 5 Sino-Africa Joint Research Centre, Chinese Academy of Sciences, Nairobi, Kenya Sino-Africa Joint Research Centre, Chinese Academy of Sciences Nairobi Kenya; 6 Jiangsu Key Laboratory for Biodiversity and Biotechnology, College of Life Sciences, Nanjing Normal University, Nanjing, China Jiangsu Key Laboratory for Biodiversity and Biotechnology, College of Life Sciences, Nanjing Normal University Nanjing China

**Keywords:** type locality, range expansion, molecular phylogeny, conspecific, sister clade

## Abstract

**Background:**

*Boulenophrys shuichengensis* (Tian & Sun, 1995) has for a long time been a subject of taxonomic ambiguity due to rare field records and a lack of molecular data from its type locality.

**New information:**

To address this, we integrated molecular, morphological and type locality survey data: three tadpoles were collected from the type locality and seven adult males from Weixin County, Yunnan Province, China. Phylogenetic analyses using concatenated 16S rRNA and *COI* sequences revealed that tadpoles from the type locality together with specimens from Weixin (Yunnan), Dafang (Guizhou) and Chongqing, formed a strongly supported monophyletic clade with no significant genetic divergence (0–0.3%). Morphological comparion further showed that both the adult specimens from Weixin and tadpoles from the type locality of *B. shuichengensis* were consistent with the original species description. Our findings: (1) provide the first validated molecular data for *B. shuichengensis* from its type locality, therefore clarifying its taxonomic status, while also offering a detailed morphological account of its tadpoles.; (2) confirm Weixin as a new Yunnan distribution record, extending the species’ range northwards by ~ 200 km; (3) verify that populations from Dafang (Guizhou) and Chongqing are conspecific, indicating a wider Yunnan–Guizhou Plateau distribution. Phylogenetically, *B. shuichengensis* is the sister taxon to *B. caudoprocta*, with both species forming a clade within the *B. boettgeri* group.

## Introduction

*Boulenophrys*, the largest genus within the subfamily Megophryinae (commonly known as horned frogs), consists of a total of 80 recognised species. It is distributed in subtropical and tropical mainland East Asia, mostly in southern China, extending south into northernmost Indochina, including Vietnam, Laos, Myanmar and Thailand (Frost 2026).

In recent decades, the genus *Boulenophrys* has attracted considerable attention due to its extensive taxonomic controversy (e.g. Dubois and Ohler (1998), Chen et al. (2017), Mahony et al. (2017), Dubios et al. (2021), Lyu et al. (2021), Qi et al. (2021)) and the substantial underestimation of its diversity (e.g. Chen et al. (2017), Lyu et al. (2021), Qi et al. (2021), Wang et al. (2022), Lyu et al. (2023)). Qi et al. (2021) reviewed the recent taxonomic literature of Megophryinae and divided the genus *Boulenophrys* into three species groups: *B. boettgeri* group, *B. minor* group, *B. omeimontis* group. *Boulenophrys fansipanensis* (Tapley, Cutajar, Mahony, Nguyen, Dau, Luong, Le, Nguyen, Nguyen, Portway, Luong and Rowley, 2018), *B. frigida* (Tapley, Cutajar, Nguyen, Portway, Mahony, Nguyen, Harding, Luong and Rowley, 2021), *B. hoanglienensis* (Tapley, Cutajar, Mahony, Nguyen, Dau, Luong, Le, Nguyen, Nguyen, Portway, Luong and Rowley, 2018) and *B. shuichengensis* (Tian & Sun, 1995) were treated as incertae sedis.

*Boulenophrys shuichengensis* was described from Fenghuang Village (now Fenghuang Subdistrict), Shuicheng County (now Zhongshan District), Liupanshui, Guizhou, China (Tian and Sun 1995). Since its description, no individuals of this species or relevant molecular data have been recorded from its type locality for several decades, leaving key gaps in understanding of its taxonomy and phylogenetic placement. Historically, based on morphological evidence (Fei and Ye 2016), *B. shuichengensis* was inferred to be closely related to *B. caudoprocta* (Shen, 1994), and then Lyu et al. (2023) placed this species within the *B. boettgeri* group following the suggestions of two anonymous reviewers. In recent years, molecular data attributed to *B. shuichengensis* have been reported from Dafang, Guizhou (approximately 150 km from its type locality) (Liu et al. 2025). However, the taxonomic authenticity of these sequences, i.e. whether they truly belong to *B. shuichengensis*, requires further verification by comparing with type locality specimens. To date, the phylogenetic placement of *B. shuichengensis* remains unresolved and awaits further clarification.

To fill these critical gaps, we conducted targeted field surveys at the type locality of *B. shuichengensis* and successfully collected tadpoles of the genus *Boulenophrys*. Additionally, a population of *Boulenophrys* species in Weixin, Zhaotong, Yunnan, China were collected, which exhibit morphological characteristics consistent with *B. shuichengensis*. The primary objectives of this study are: (1) to clarify the taxonomic status of *B. shuichengensis* using an integrative approach, leveraging the first genetic data collected from its type locality; (2) to formally report a new distribution record of *B. shuichengensis* in Yunnan, thus expanding our understanding of its geographic range.

## Materials and methods

### Sampling

**Figure 1.**
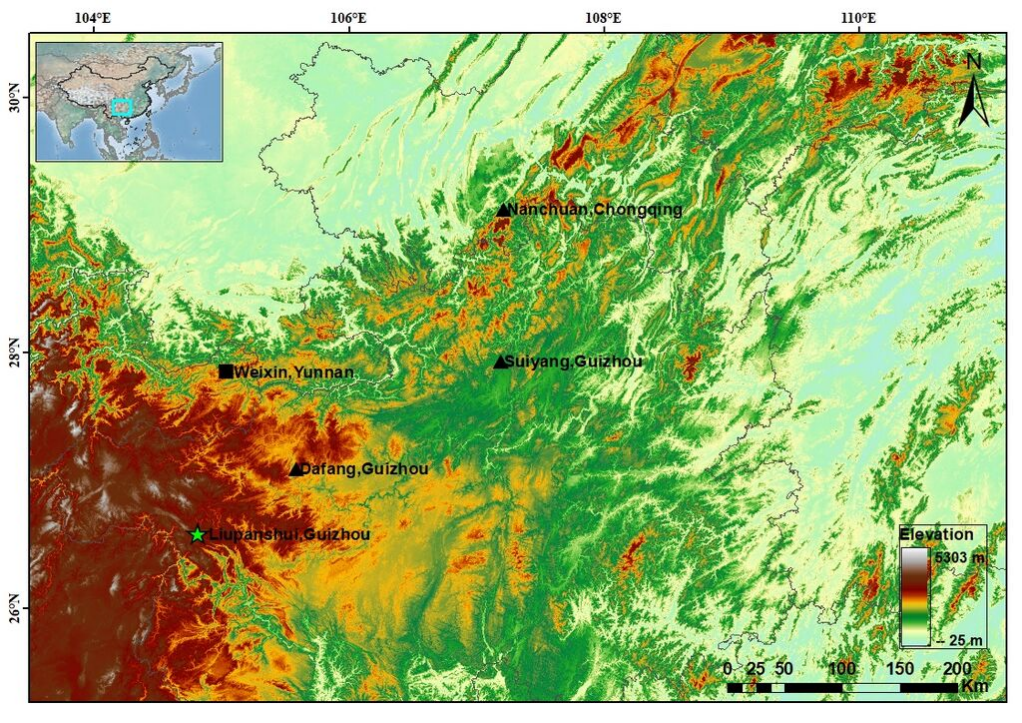
Distribution records of *Boulenophrys shuichengensis*, the green star indicating the type locality.

Field surveys were conducted at two localities: 1) Fenghuang Subdistrict (26.5810°N, 104.8066°E; 1835 metres above sea level, m a.s.l.), Zhongshan District, Liupanshui City, Guizhou Province, China, in September 2023; and 2) Weixin County (27.8519–27.8540°N, 105.0336–105.0369°E; 1387–1414 m a.s.l.), Zhaotong City, Yunnan Province, China, in June 2024 (Fig. 1). From the former locality, three tadpoles were sampled, while seven adult male *Boulenophrys* specimens were collected and photographed from the latter. All specimens were euthanised and fixed in 75% ethanol for permanent preservation. Tails or liver tissue samples were preserved in 95% ethanol for molecular analyses. The specimens and tissue samples were deposited in the Museum of Herpetology, Nanjing Normal University (NNU), Nanjing, China. All research protocols were approved by the Institutional Animal Care and Use Committee (IACUC) of Nanjing Normal University (approval number: IACUC-2025068).

### DNA extraction, PCR and sequencing

Genomic DNA was extracted from tails or liver tissues using the FastPure Cell/Tissue DNA Isolation Mini Kit (Vazyme, Nanjing, China). The mitochondrial 16S ribosomal RNA (16S rRNA) and cytochrome c oxidase subunit I (*COI*) genes were amplified and sequenced for ten specimens using the primer pairs 16S rRNA-F (5'–CGCCTGTTTAYCAAAAACAT–3') and 16S rRNA-R (5'–CCGGTYTGAACTCAGATCAYGT–3') (Kocher et al. 1989), as well as Chmf4 (5′–TYTCWACWAAYCAYAAAGAYATCGG–3′) and Chmr4 (5′– ACYTCRGGRTGRCCRAARAATCA–3′) (Che et al. 2012). PCR amplifications were performed in a 25 μl reaction volume with the following conditions: initial denaturing step at 95°C for 4 min, 35 cycles of denaturing at 94°C for 40 s, annealing at 55°C for 16S rRNA and COI for 1 min and extending at 72°C for 1 min and a final extension at 72°C for 10 min. The amplified PCR product was purified using the Qiagen PCR purification kit and sequences in both directions were obtained from an ABI 3100 automated sequencer at Kunming TSING KE Biological Technology Co. Ltd. (Kunming, China). New sequences were deposited in GenBank (the GenBank accession numbers are available in Suppl. material 1). Newly-obtained sequences were first assembled and edited using DNASTAR LASERGENE 7.1 (Burland 2000).

### Phylogenetic analyses

A total of 185 sequences (16S rRNA and *COI*) were used for the molecular analyses, representing 77 species of the genus *Boulenophrys*. Twenty sequences were derived from tail or liver tissue samples collected in this study and 165 homologous sequences were downloaded from GenBank (Suppl. material 1). Following Chen et al. (2017), *Xenophrys mangshanensis* and *X. glandulosa* were used as outgroups in this study. All sequences were assembled and aligned using MUSCLE 3.8 with default settings (Edgar 2004) and then visually checked for accuracy and trimmed to minimise missing characters in MEGA 6 (Tamura et al. 2013). Based on the concatenated 16S rRNA and *COI* dataset, phylogenetic analyses were conducted using Bayesian Inference (BI) and Maximum Likelihood (ML) methods, both implemented in PHYLOSUITE (Zhang et al. 2020). The best-fit substitution model was selected under the Bayesian Information Criterion (BIC) by the programme PARTITION FINDER 2 (Lanfear et al. 2012). The GTR + I + G model was chosen as the best-fit model following the Bayesian Information Criterion (BIC) for both 16S rRNA and *COI*. For BI analysis, the Monte Carlo Markov chain length was run for 10 million generations with sampling every 1000 generations, discarding the first 25% of trees as the “burn-in”. For ML analysis, nodal supports were estimated from 1000 bootstrap replicates under the GTR+Gamma model. Mean genetic distances between and within species were calculated using uncorrected pairwise distances (*p*-distance) by 16S rRNA implemented in MEGA v.6.0.6, with complete deletion of missing data and gaps (Tamura et al. 2013).

### Morphological analyses

The seven preserved adult specimens were measured with digital calipers to the nearest 0.1 mm. Measurements followed Fei et al. (2009). Measurements included the following: SVL (Snout-vent length): measured from tip of snout to vent; HDL (head length): measured from tip of snout to jaw angle; HDW (head width): measured as head width at its widest point; SNT (snout length): measured from tip of snout to anterior corner of eye; INS (internasal space): measured as distance between nares; IOD (interorbital space): measured at narrowest point between eyelids on top of head; NED (nasal to eye distance): measured as distance from the anterior corner of eye to nostril centre; UEW (upper eyelid width): maximum width of upper eyelid; ED (eye diameter): measured as the distance between corners of eye; TD (tympanum diameter): measured as maximal diameter of tympanum; TEY: distance from anterior border of tympanum to posterior orbital border; SN: distance from the centre of the nostril to the tip of the snout; IFE (internal front of eyes): the shortest distance between the anterior orbital borders of the eyes; internal back of eyes (IBE): the shortest distance between the posterior orbital borders of the eyes; forearm length (FAL): measured from the elbow to the wrist; HND (hand length): measured as the distance from the proximal edge of inner metacarpal tubercle to the tip of third finger; FEM (femoral length): measured from the cloaca to the knee; TIB (tibia length): measured as the distance from knee to heel; TL (Length of tarsus); FTL (foot length): measured as the distance from proximal end of inner metatarsal tubercle to the tip of fourth toe; IMT (length of inner metatarsal tubercle). Sex was determined by examination of the gonads.

The tadpole specimens were also measured with digital calipers to the nearest 0.1 mm. The staging followed Gosner (1960). According to Fei et al. (2009), the following morphometric measurements of tadpoles were taken: total length (TL, from the tip of the snout to the tip of the tail), snout-vent length (SVL, from the tip of the snout to the base of the anal tube), body height (BH, maximum height between the dorsal and ventral surfaces of the body), body width (BW, maximum width of the body), snout to spiraculum (SS, from the tip of the snout to the opening of the spiraculum), interocular distance (IOS, minimum distance between the two eyes), tail length (TAL, from the base of the anal tube to the tip of the tail), tail height (TH, maximum height between the upper and lower margins of the tail) and tail muscle diameter (TMD, maximum diameter at the base of the tail).

## Taxon treatments

### Boulenophrys shuichengensis

(Tian & Sun, 1995)

6C89248F-7A9C-58CC-A14B-FE7190883316

#### Materials

- **Type status:** Other material. **Occurrence:** catalogNumber: T2366; recordedBy: Xiong Rong-Chuan; individualCount: 1; sex: unavailable; lifeStage: tadpole; occurrenceID: C07C2036-D84F-5874-BE1A-1D5141C94E61; **Location:** country: China; stateProvince: Guizhou; municipality: Liupanshui; locality: Fenghuang subdistrict, Zhongshan District; verbatimElevation: 1835 m; decimalLatitude: 26.581; decimalLongitude: 104.8066; **Event:** year: 2023; month: 9; day: 11; **Record Level:** basisOfRecord: PreservedSpecimen
- **Type status:** Other material. **Occurrence:** catalogNumber: T2367; recordedBy: Xiong Rong-Chuan; individualCount: 1; sex: unavailable; lifeStage: tadpole; occurrenceID: 437B5A12-0597-5690-B2C2-B326DC85C3B0; **Location:** country: China; stateProvince: Guizhou; municipality: Liupanshui; locality: Fenghuang Subdistrict, Zhongshan District; verbatimElevation: 1835 m; decimalLatitude: 26.581; decimalLongitude: 104.8066; **Event:** year: 2023; month: 9; day: 11; **Record Level:** basisOfRecord: PreservedSpecimen
- **Type status:** Other material. **Occurrence:** catalogNumber: T2368; recordedBy: Xiong Rong-Chuan; individualCount: 1; sex: unavailable; lifeStage: tadpole; occurrenceID: 68D57A03-2AC6-5870-B888-7545E9CC4540; **Location:** country: China; stateProvince: Guizhou; municipality: Liupanshui; locality: Fenghuang Subdistrict, Zhongshan District; verbatimElevation: 1835 m; decimalLatitude: 26.581; decimalLongitude: 104.8066; **Event:** year: 2023; month: 9; day: 11; **Record Level:** basisOfRecord: PreservedSpecimen
- **Type status:** Other material. **Occurrence:** catalogNumber: NNU040609; recordedBy: Chen Lu; individualCount: 1; sex: male; lifeStage: adult; occurrenceID: 878843E1-D61C-5F5B-B58C-D539C628E050; **Location:** country: China; stateProvince: Yunnan; municipality: Zhaotong; locality: Weixin County, Zaxi Town; verbatimElevation: 1387–1414 m; decimalLatitude: 27.854; decimalLongitude: 105.0369; **Event:** eventRemarks: collected on 10 July 2024 by Lu Chen; **Record Level:** basisOfRecord: PreservedSpecimen
- **Type status:** Other material. **Occurrence:** catalogNumber: NNU040610; recordedBy: Chen Lu; individualCount: 1; sex: male; lifeStage: adult; occurrenceID: C8BD25CF-93A3-5DD0-8E92-23419706F497; **Location:** country: China; stateProvince: Yunnan; municipality: Zhaotong; locality: Weixin County, Zaxi Town; verbatimElevation: 1387–1414 m; decimalLatitude: 28.854; decimalLongitude: 106.0369; **Event:** eventRemarks: collected on 10 July 2024 by Lu Chen; **Record Level:** basisOfRecord: PreservedSpecimen
- **Type status:** Other material. **Occurrence:** catalogNumber: NNU040611; recordedBy: Chen Lu; individualCount: 1; sex: male; lifeStage: adult; occurrenceID: 151B2D09-F9E0-5DAF-B34E-C1319ADC5CE8; **Location:** country: China; stateProvince: Yunnan; municipality: Zhaotong; locality: Weixin County, Zaxi Town; verbatimElevation: 1387–1414 m; decimalLatitude: 29.854; decimalLongitude: 107.0369; **Event:** eventRemarks: collected on 10 July 2024 by Lu Chen; **Record Level:** basisOfRecord: PreservedSpecimen
- **Type status:** Other material. **Occurrence:** catalogNumber: NNU040612; recordedBy: Chen Lu; individualCount: 1; sex: male; lifeStage: adult; occurrenceID: 38060A03-E5A0-5636-BB44-6C5A410A6DB3; **Location:** country: China; stateProvince: Yunnan; municipality: Zhaotong; locality: Weixin County, Zaxi Town; verbatimElevation: 1387–1414 m; decimalLatitude: 30.854; decimalLongitude: 108.0369; **Event:** eventRemarks: collected on 10 July 2024 by Lu Chen; **Record Level:** basisOfRecord: PreservedSpecimen
- **Type status:** Other material. **Occurrence:** catalogNumber: NNU040613; recordedBy: Chen Lu; individualCount: 1; sex: male; lifeStage: adult; occurrenceID: B86BF828-4C43-5F7F-A22D-098ED2493F9D; **Location:** country: China; stateProvince: Yunnan; municipality: Zhaotong; locality: Weixin County, Zaxi Town; verbatimElevation: 1387–1414 m; decimalLatitude: 31.854; decimalLongitude: 109.0369; **Event:** eventRemarks: collected on 10 July 2024 by Lu Chen; **Record Level:** basisOfRecord: PreservedSpecimen
- **Type status:** Other material. **Occurrence:** catalogNumber: NNU040619; recordedBy: Chen Lu; individualCount: 1; sex: male; lifeStage: adult; occurrenceID: DE7F4835-4E1F-527E-BC19-098A88F99FEA; **Location:** country: China; stateProvince: Yunnan; municipality: Zhaotong; locality: Weixin County, Zaxi Town; verbatimElevation: 1387–1414 m; decimalLatitude: 27.8519; decimalLongitude: 105.0336; **Event:** eventRemarks: collected on 10 July 2024 by Lu Chen; **Record Level:** basisOfRecord: PreservedSpecimen
- **Type status:** Other material. **Occurrence:** catalogNumber: NNU040620; recordedBy: Chen Lu; individualCount: 1; sex: male; lifeStage: adult; occurrenceID: 8D86BD3E-7617-56D4-A922-3319CC810CE9; **Location:** country: China; stateProvince: Yunnan; municipality: Zhaotong; locality: Weixin County, Zaxi Town; verbatimElevation: 1387–1414 m; decimalLatitude: 27.8519; decimalLongitude: 105.0336; **Event:** eventRemarks: collected on 10 July 2024 by Lu Chen; **Record Level:** basisOfRecord: PreservedSpecimen

#### Description

##### Morphological description (measurements in mm)

See Table 1. Body size large, adult male with SVL 66.7–89.0 mm (n = 7); head length (HDL 20.5–24.5 mm, 27.1%–30.7% of SVL) slightly equal to the head width (HW 23.2–27.6 mm, 30.5–34.8% of SVL); snout slightly obtusely rounded in dorsal view, projecting beyond lower jaw; nostril rounded, laterally orientated, lying near the mid-line between the snout tip and eyes; canthus rostralis distinct; loreal region concave; snout (SNT 7.7–9.9 mm, 9.7–11.5% of SVL) shorter than eye diameter (ED 8.6–11.7 mm, 11.0–14.2% of SVL); width of upper eyelid (UEW 8.2–9.9 mm) larger than internasal distance (INS 6.3–7.6 mm) and interorbital distance (IOD 5.4–7.7 mm); eye diameter 105.1–133.0% times that of SNT; pupil vertical, near diamond-shaped; tympanum distinct, its length nearly half eye diameter; vomerine ridge present, “\ /”-shape, vomerine teeth absent; maxillary teeth present; iris golden brown; tongue heart-shaped, slightly notched posteriorly.

**Figure 2.**
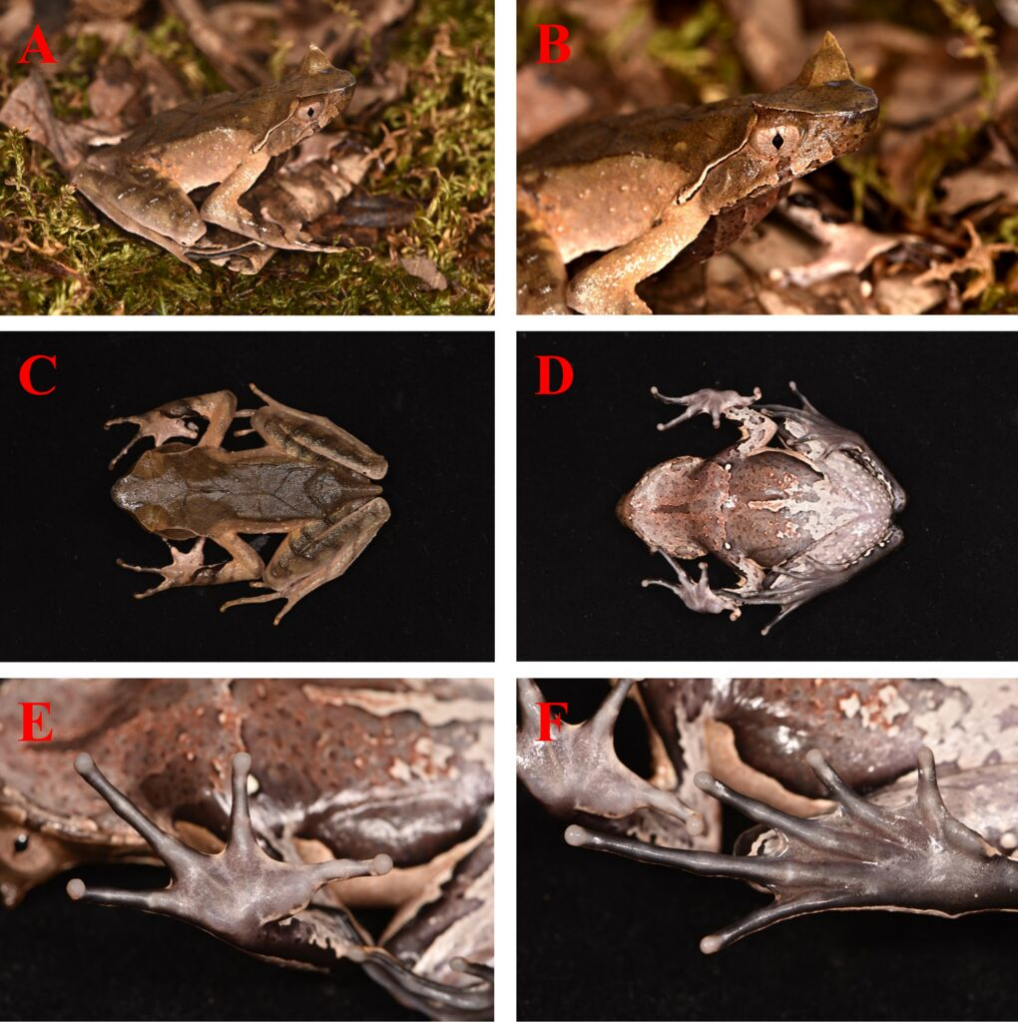
*Boulenophrys shuichengensis* (NNU 040619). Lateral view (A), lateral view of the head (B), dorsal view (C), ventral view of thighs (D), ventral view of hand (E) and foot (F). Photos by Yun-He Wu.

Fore-limbs slender; fore-limb length (FAL 17.3–20.6 mm, 23.1%–26.6% of SVL) longer than hand length (HND 5.5–8.1 mm, 8.2%–9.9% of SVL); relative length of fingers: II < I < IV < III; tips of all fingers slightly dilated, round; terminal grooves absent; fingers with lateral dermal fringes; hand lacking webbing; subarticular tubercles indistinct, large, near the palm smaller, formula: 1, 1, 2, 1; two metacarpal tubercles, inner metacarpal tubercle large, oval, outer metacarpal tubercle enlongated (Fig. 2).

Hind-limbs short, foot shorter than tibia, tibia length (TL) 64.1–78.1% of SVL, foot length (FTL) 40.9–49.2% of SVL; tibial-tarsal articulation beyond the anterior margin of the eye or reaches the mid-point of the eye when hind-limb is adpressed along the side of the body; the heels overlapping when legs are held at right angles to the body axis; relative length of toes: I < II < V < III < IV; tips of toes round and slightly dilated; toe webbing distinct, about rudimentary webbing; toes with wide lateral dermal fringes; subarticular tubercles indistinct; inner metatarsal tubercle greatly enlarged (IMT 5.4–7.0 mm, 6.1%–9.5% of SVL), outer metatarsal tubercle absent.

Dorsal skin relatively rough, with scattered numerous granules, tubercles and dermal ridges; single horn-like tubercle at the edge of each upper eyelid very prominent; two opposing "V"-shaped glandular skin folds present on dorsum joined by an ca. 10–15 mm long dorsomedial fold in a hourglass-shape; two discontinuous dorsolateral parallel ridges on either side of the hourglass-shape ridges; obvious supratympanic fold curving ventrally from posterior corner of eye to level above insertion of arm, slightly widening posteriorly; flanks with some tubercles; dorsal surface of limbs with small tubercles forming distinct transverse skin folds; ventral surfaces of limbs, chest, abdomen and throat smooth; pectoral gland large, closer to axilla; femoral glands distinct, closer to knee than to vent.

Males lack nuptial pads, nuptial spines and vocal sacs.

##### Colour in life

Dorsum yellowish-brown with a complete dark brown triangle between the eyes; three dark brown vertical bars on upper lip; iris light-brown with tiny dark reticulations spreading from pupil; supratympanic fold with a light edge; ventral surfaces of body and limbs primarily white to grey, mottled with dense black and white blotches on belly, surface of throat with three black longitudinal band, middle one distinctly longer; tips of digits, subarticular tubercles, metacarpal and metatarsal tubercles greyish-white; pectoral glands and femoral glands white.

##### Tadpoles

**Figure 3.**
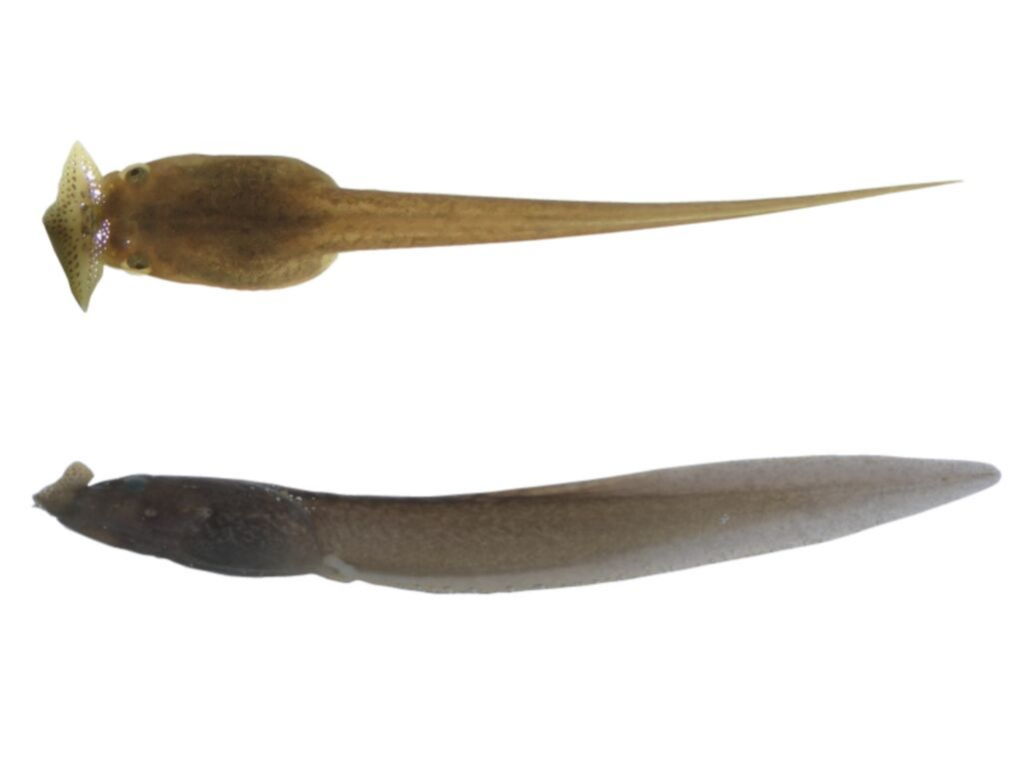
*Boulenophrys shuichengensis* tadpole.

Tadpoles collected in September measured 37.2–40.6 mm in total length and 11.7–12.7 mm in snout–vent length (n = 3) (Table 2, Fig. 3). At Gosner Stages 31–35, tadpoles possessed a single pair of hind limbs; body slender, light brown; eyes dorsolateral with round pupils; nares round, laterally orientated, with rims raised from the body wall and directed laterally; spiracle sinistral, positioned low on the left flank; spiracular tube short, projecting posterodorsally, free at the tip from the body, with a posterolateral opening; anal tube medially opening, situated at the base of the ventral caudal fin; dorsal and ventral caudal fins originating from the trunk; tail muscle robust; tail tip bluntly pointed, with brownish-black spots present on the fins; mouth terminal, oral disc forming a rhomboidal funnel bearing radiating labial papillae.

#### Distribution

Currently, *B. shuichengensis* in Yunnan Province has only been recorded in the mountains around Zhaxi Town, Weixin County, at an altitude of approximately 1400 metres. The collection site was near mountain streams 1.5–2 metres wide, surrounded by subtropical evergreen broad-leaved forests, coniferous and broad-leaved mixed forests and bamboo forests. The stream water was cold and clear, with muddy or rocky substrates, a steep gradient and a potential groundwater source at the headwaters. The banks were covered with fallen leaves, calamus and other herbaceous plants. Most specimens were collected under fir forests about 150 metres from the stream, where the ground was covered with thick leaf litter and lacked understorey shrubs and herbaceous plants. During field surveys in mid- to late June of 2024, no courtship behaviour, amplexus or oviposition was observed and no vocalisations were heard. However, when captured and held in hand, adult toads rapidly vibrated their throats, producing faint, rapid sounds. A village is located about 1 km from the collection site. *Leptobrachella weixinensis*, *Nanorana yunnanensis* and *Bufo andrewsi* were present in the surrounding area, and *Oreolalax rhodostigmatus* was found co-existing with this species.

Tadpoles were collected from the type locality of *B. shuichengensis* (Fenghuang Subdistrict, Zhongshan District, Liupanshui City, Guizhou Province, China) at an elevation of ~ 1900 m. This region features abundant rainfall year-round and a subtropical monsoon humid climate, with a mean annual temperature of 15°C. The sampling site was a slow-flowing stream section (2–4 metres wide), near the bank with a muddy or sandy substrate and numerous flat limestone rocks. The stream was fed by surface flow and groundwater into a large reservoir. The banks were covered with typical subtropical evergreen broad-leaved forests, characterised by limestone karst terrain, alongside dense shrubs and herbaceous vegetation. During the rainy season, water levels rise markedly and the water turns turbid, with significant fluctuations tied to precipitation. An urban area and a school are located approximately 1.5 km from the collection site. Amphibian species documented in the same area include *Pseudohynobius shuichengensis*, *Odorrana grahami*, *Rana chaochiaoensis*, *Bufo andrewsi* and *Oreolalax rhodostigmatus*.

The tadpoles were collected at night in slow-flowing areas of the stream, moving slowly along the water surface. When exposed to light, they immediately hid under nearby rocks and sediment. Despite repeated field surveys in Zhongshan District, Liupanshui City from 2023 to 2025, no adult toads were found.

## Analysis

**Figure 4.**
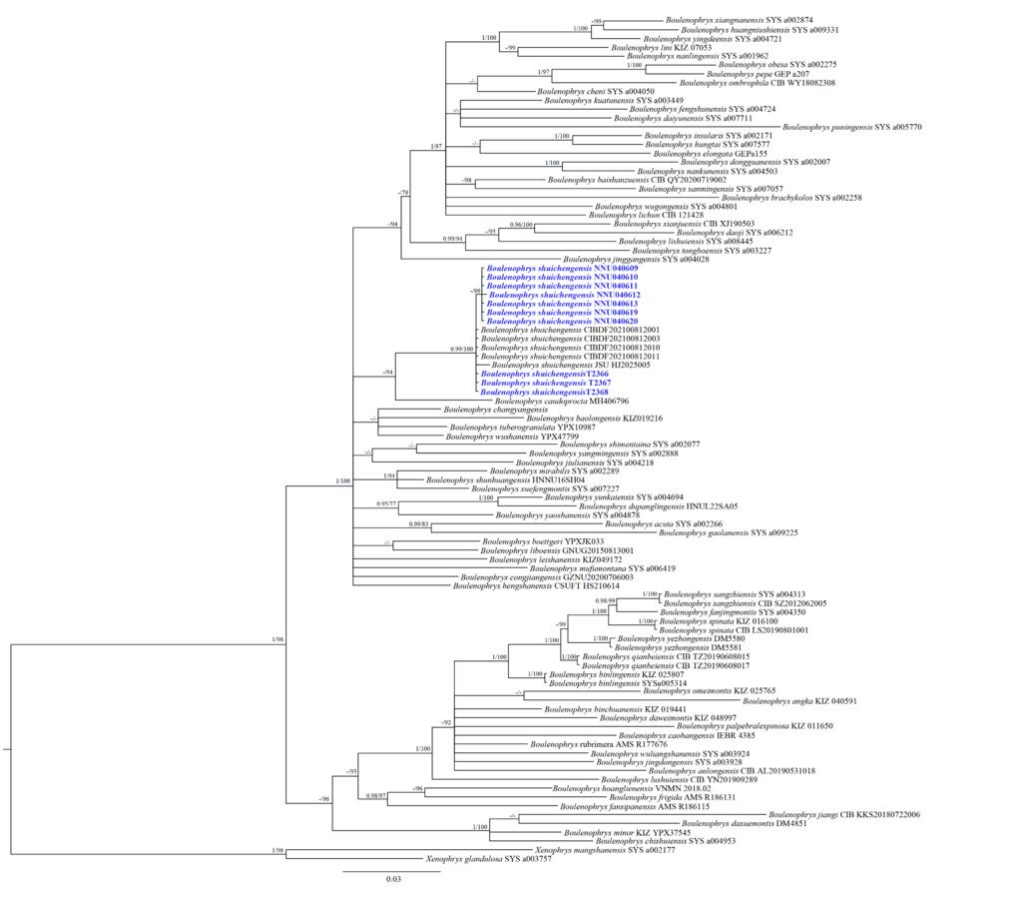
Bayesian Inference (BI) tree of the genus *Boulenophrys* reconstructed based on the 16S rRNA and *COI* gene sequences. The newly-obtained sequences in this study are indicated in blue. Bayesian posterior probability (BPP)/ML bootstrap supports (BS) are denoted beside each node.

A total of 537 base pairs (bp) of the 16S rRNA gene and 561 bp of the *COI* gene were concatenated into a 1,098-bp sequence matrix. ML and BI phylogenetic analyses yielded essentially identical topologies, with the BI phylogenetic topology shown in Fig. 4. All *Boulenophrys* samples formed a well-supported monophyletic clade (BI = 1.00 and ML = 98). Furthermore, tadpoles from the type locality, alongside adult specimens from the newly-discovered Weixin population (Zhaotong City, Yunnan Province), Dafang County (Guizhou Province) and Chongqing Municipality, clustered into a strongly supported monophyletic lineage (BS = 100; BPP = 0.99). No significant genetic divergence was detected amongst these samples (0–0.3%; see Suppl. material 2). In addition, this lineage was recovered as the sister taxon of *B. caudoprocta* with moderate statistical support (BS = 94; BPP = 0.81), which was not labelled on the tree as the posterior probability fell below the 0.95 annotation threshold. The genetic divergence between the two lineages was 3.4%.

Morphologically, the adult specimens from Weixin were consistent with the original diagnosis of the type series (Tian et al. 2000), characterised by the presence of a vomerine ridge, absence of vomerine teeth, a single prominent horn-like tubercle on the lateral edge of each upper eyelid and males lacking nuptial pads/spines as well as vocal sacs. Additionally, the tadpoles from the type locality of *B. shuichengensis* matched the historical descriptions (Tian and Sun 1995, Tian et al. 2000, Fei et al. 2009), featuring a slender light-brown body, a rhomboidal oral disc with radiating labial papillae, a sinistral spiracle, a medially orientated anal tube and a bluntly pointed tail fin marked with brownish-black spots.

## Discussion

Since its initial description (Tian and Sun 1995), *B. shuichengensis* has been plagued by taxonomic uncertainty due to rare field records and the absence of molecular data from its type locality (Qi et al. 2021, Lyu et al. 2023). Our study fills these critical gaps through complementary lines of evidence.

Historical records and our extensive targeted surveys (weekly night-time surveys were performed from March to July in 1994 and 1995, additional surveys were conducted sporadically in July and September of 2024–2025) confirm that , *B. shuichengensis* is the sole Megophryinae species documented at the type locality (Fenghuang Subdistrict, Zhongshan District, Liupanshui, Guizhou) (Tian and Sun 1995, Chen et al. 2013and personal survey). In addition, the tadpoles of Megophryinae species collected from the type locality in September 2023 exhibited morphological features fully consistent with the original description of this species, including oral disc structure, body shape, spiracle and anal tube. These findings validate the molecular sequences obtained in this study, serving as a long-missing key resource to resolve the taxonomic status of *B. shuichengensis*. However, to date, no adult individuals of *B. shuichengensis* from its type locality have been documented since its original description. Targeted field surveys are recommended in future to obtain comprehensive life history data, thereby providing robust scientific support for the conservation of this poorly-known amphibian species.

Molecular phylogenetic analyses revealed that the newly-discovered Weixin population (Zhaotong City, Yunnan Province), along with samples from Dafang County (Guizhou Province), Chongqing Municipality and the type locality tadpoles, form a strongly supported clade (BS = 100; BPP = 0.99). No genetic divergence was observed amongst these samples (0–0.3%), confirming genetic homogeneity across the populations. Morphologically, adult specimens from Weixin exhibit strong congruence with the original description of *B. shuichengensis* (Tian et al. 2000), particularly in diagnostic characters matching the holotype. However, a notable difference was obvserved in body size: male specimens from Weixin had a snout‑vent length (SVL) ranging from 66.7 to 89.0 mm, which was significantly smaller than the male holotypes (SVL > 100 mm). Gonadal examination verified that all dissected males possessed mature testes, confirming these specimens were sexually mature adults. The observed body size difference is likely attributable to two factors (Sebens 1987): (1) Indeterminate growth in amphibians, whereby adults may continue to grow somatic tissues post-sexual maturity, resulting in larger body sizes in older individuals; (2) Inherent inter-population morphological differentiation between the type locality and the Weixin population.

This concordance between morphological consistency and genetic monophyly unambiguously confirms three key findings: (1) The Weixin population represents a new distribution record for *B. shuichengensis* in Yunnan Province; (2) Samples from Dafang and Chongqing are conspecific with *B. shuichengensis*; and (3) the species is not endemic to its type locality. This discovery marks an approximately 200 km northwards range expansion, suggesting that *B. shuichengensis* may be more widely distributed across the Yunnan-Guizhou Plateau than previously hypothesised. Undocumented populations may, therefore, exist in intervening montane areas (e.g. north-eastern Yunnan, western Guizhou) that remain undersurveyed. Notably, this species were reported from Suiyang County, Guizhou Province, with textual and photographic evidence (Fei et al. 2009, Fei et al. 2012 and Fei and Ye 2016). Further examination of museum specimens and targeted field surveys in Suiyang County are needed to verify this record. Additionally, the apparent fragmentation of known populations highlights the need for targeted surveys to elucidate the species’ complete distribution range.

Phylogenetic analyses further indicate that *B. shuichengensis* is most closely related to *B. caudoprocta* (Fig. 4), consistent with earlier morphological inference (Fei and Ye 2016). Together, these two species form a moderately supported monophyletic clade within the *B. boettgeri* group (BS = 94; BPP = 0.81, not labelled on the tree due to a posterior probability < 0.95), corroborating Lyu et al. (2023) preliminary placement of *B. shuichengensis* in this group.

Beyond phylogenetic and distributional insights, our study resolves a critical morphological discrepancy in the species’ description. Tian and Sun (1995) originally reported that adult male *B. shuichengensis* possess a subgular vocal sac; however, Tian et al. (2000) subsequently re-described the species using the same holotype, stating males “lack subgular vocal sacs”, a direct contradiction. Our detailed examination of adult males from the Weixin population confirms that males lack a subgular vocal sac, consistent with the re-description by Tian et al. (2000). This discrepancy may stem from misobservation in the original description and our findings clarify this key diagnostic character for future species identification. Notably, this characteristic is also shared with *B. caudoprocta*. Furthermore, as the males of this species lack secondary sexual characteristics, such as vocal sacs, nuptial spines and nuptial pads, distinguishing between sexes in the field is challenging. Sex can only be reliably determined by dissecting collected specimens and examining the gonads in a laboratory setting.

To date, our understanding of the natural history of *B. shuichengensis* remains considerably limited. The reproductive season and breeding habits of this species are still unclear (Fei et al. 2012). However, males lack vocal sacs, as in *B. caudoprocta*; this undoubtedly complicates efforts to locate this species. Furthermore, field surveys conducted between 2023 and 2025 failed to observe egg masses, amplexus or courtship behaviour of this species in the field.

The closely-related species *B. caudoprocta* was recorded breeding in cave groundwater in Xianfeng, Hubei, where its tadpoles share the same subterranean cave environment with *Odorrana wuchuanensis*. Similar to *B. shuichengensis*, male *B. caudoprocta* lack vocal sacs and do not vocalise during the breeding season; they reproduce in August within mountainous streams, characterised by dense forest canopy, clear water, steep gradients and relatively fast-flowing currents (Shen et al. 2013). Whether *B. shuichengensis* exhibits similar habits requires further investigation.

## Supplementary Material

XML Treatment for Boulenophrys shuichengensis

385CE619-D335-5FEA-B964-21E5DB12F35E 10.3897/BDJ.14.e188165.suppl1 Supplementary material 1 Table S1 Data type Samples Brief description Table S1. Samples used in molecular phylogenetic analyses in this study. File: oo_1585121.xlsx https://binary.pensoft.net/file/1585121 Dongru Zhang, Lu Chen, Rongchuan Xiong, Wenjing Shen, Felista Kasyoka Kilunda, Miao Zhong, Weiwei Pan, Jianhong Li, Yunhe Wu

55B3F54C-E7FE-5330-9D46-60E5591BCEB7 10.3897/BDJ.14.e188165.suppl2 Supplementary material 2 Table S2 Data type genetic distance Brief description Genetic distance of 16s sequences File: oo_1533770.xls https://binary.pensoft.net/file/1533770 Dongru Zhang, Lu Chen, Rongchuan Xiong, Wenjing Shen, Felista Kasyoka Kilunda, Weiwei Pan, Miao Zhong, Jianhong Li, Yunhe Wu

## Figures and Tables

**Table 1.** Measurements (mm) of *Boulenophrys shuichengensis*.

	NNU 040620	NNU 040619	NNU 040613	NNU 040612	NNU 040611	NNU 040610	NNU 040609
Sex	♂	♂	♂	♂	♂	♂	♂
SVL	73.4	66.7	82.4	89.0	85.7	77.0	81.1
HDL	22.3	20.5	22.3	24.5	24.3	22.2	22.5
HDL/SVL	30.4%	30.7%	27.1%	27.5%	28.4%	28.8%	27.7%
HDW	25.4	23.2	25.4	27.6	26.4	25.9	24.7
HDW/SVL	34.6%	34.8%	30.8%	31.0%	30.8%	33.6%	30.5%
SNT	7.8	7.7	8.8	9.9	8.3	8.8	8.4
SNT/SVL	10.6%	11.5%	10.7%	11.1%	9.7%	11.4%	10.4%
INS	6.3	7.0	6.9	7.4	7.6	6.7	7.3
IOD	6.6	5.4	6.4	6.7	7.7	6.5	6.9
UEW	8.8	8.2	9.9	9.9	9.3	9.3	8.8
ED	10.1	8.6	11.7	10.4	10.4	10.4	8.9
ED/SNT	129.5%	111.7%	133.0%	105.1%	125.3%	118.2%	106.0%
ED/SVL	13.8%	12.9%	14.2%	11.7%	12.1%	13.5%	11.0%
TD	4.3	4.3	4.6	3.9	4.6	4.3	4.5
TD/ED	42.6%	50.0%	39.3%	37.5%	44.2%	41.3%	50.6%
TEY	4.3	4.8	5.1	5.3	5.9	5.1	4.8
SN	4.8	4.4	5.2	5.28	5.5	5.0	5.0
NED	3.5	3.6	4.3	4.5	4.2	4.2	4.4
IFE	11.3	10.5	12.2	12.9	12.5	12.2	11.9
IBE	19.3	17.5	20.2	21.1	20.2	20.1	19.3
FAL	19.5	17.3	19.5	20.6	20.3	19.5	19.7
FAL/SVL	26.6%	25.9%	23.7%	23.1%	23.7%	25.3%	24.3%
HND	7.3	5.5	7.2	8.1	8.1	7.4	7.6
HND/SVL	9.9%	8.2%	8.7%	9.1%	9.5%	9.6%	9.4%
FEM	37.6	34.5	37.9	40.3	40.5	37.5	38.2
TIB	38.2	34.4	36.1	38.0	39.7	37.9	38.0
TL	55.7	52.1	54.2	57.0	56.6	54.4	56.3
TL/SVL	75.9%	78.1%	65.8%	64.1%	66.0%	70.6%	69.4%
FTL	34.7	32.8	34.6	36.4	36.8	34.1	34.1
FTL/SVL	47.3%	49.2%	42.0%	40.9%	42.9%	44.3%	42.0%
IMT	7.0	5.7	5.5	5.4	6.2	5.9	6.2
IMT/SVL	9.5%	8.5%	6.7%	6.1%	7.2%	7.7%	7.6%
FIL	9.7	10.2	9.9	10.4	9.6	9.5	10.2
FIIL	8.8	9.3	9.1	8.7	8.6	8.8	9.6
FIIIL	15.4	13.4	13.5	13.5	13.7	13.3	13.2
FIVL	11.2	10.4	10.3	10.6	10.8	10.0	11.0
TIL	14.3	11.7	14.0	14.6	14.5	12.3	12.9
TIIL	19.3	17.0	18.9	19.5	19.4	16.7	17.4
TIIIL	27.0	24.3	25.2	26.6	26.4	24.4	23.9
TIVL	36.1	33.8	34.6	36.8	35.7	33.7	33.5
TVL	25.8	23.5	24.3	26.0	24.7	23.0	23.8

**Table 2.** Tadpoles measurements (mm) of *Boulenophrys shuichengensis*.

Voucher No.	T2366	T2367	T2368
Stage	35	35	31
TL	40.63	39.76	37.22
SVL	12.68	12.35	11.71
BH	4.59	4.51	4.48
BW	5.42	5.44	5.36
SS	6.35	7.20	6.22
IOS	3.58	3.60	2.30
TL	27.89	26.59	25.20
TH	5.75	5.56	5.17
TMD	3.28	3.10	2.71
